# Acceptability of mentor mother peer support for women living with HIV in North-Central Nigeria: a qualitative study

**DOI:** 10.1186/s12884-021-04002-1

**Published:** 2021-08-07

**Authors:** Angela Odiachi, Maryam Al-Mujtaba, Nguavese Torbunde, Salome Erekaha, Abayomi J. Afe, Ebun Adejuyigbe, Hadiza S. Galadanci, Tongdiyen L. Jasper, Llewellyn J. Cornelius, Nadia A. Sam-Agudu

**Affiliations:** 1 Research Consultant, Abuja, Nigeria; 2 International Research Center of Excellence, Institute of Human Virology Nigeria, Abuja, Nigeria; 3 Present address: Duke University School of Nursing, Durham, USA; 4 Pediatric and Adolescent HIV Unit, Prevention, Care and Treatment Department, Institute of Human Virology Nigeria, Abuja, Nigeria; 5 Present address: Elizabeth Glaser Pediatric AIDS Foundation, Abuja, Nigeria; 6 Present address: Society for Family Health, Abuja, Nigeria; 7 Department of Community Medicine, Equitable Health Access Initiative, Lagos, Nigeria; 8 Department of Pediatrics, Faculty of Clinical Sciences, Obafemi Awolowo University, Ile-Ife, Nigeria; 9 Africa Center of Excellence for Population Health and Policy, Bayero University, Kano, Nigeria; 10 Continuous Quality Improvement Unit, Learning and Development Department, Institute of Human Virology Nigeria, Abuja, Nigeria; 11 School of Social Work and College of Public Health, University of Georgia Athens, Athens, GA USA; 12 Institute of Human Virology and Department of Pediatrics, University of Maryland School of Medicine, Baltimore, USA

**Keywords:** Mentor mothers, Expert mothers, Peer support, Counseling, HIV, PMTCT, Nigeria

## Abstract

**Background:**

Mentor mothers provide psychosocial and other support to pregnant and post-partum women living with HIV (WLHIV), which has been shown to enhance maternal-infant outcomes in the prevention of mother-to-child transmission of HIV (PMTCT). Our objective was to assess the acceptability of mentor mothers as a PMTCT intervention, and to explore opinions on mentor mother program composition and delivery among stakeholders in North-Central Nigeria.

**Methods:**

We conducted nine focus group discussions and 31 in-depth interviews with 118 participants, including WLHIV, pregnant women, male partners, health workers, traditional birth attendants, community leaders, PMTCT program implementers, and policymakers. Participants were purposively recruited from health facilities and surrounding communities in the Federal Capital Territory and Nasarawa State. Transcripts were manually analysed using a Grounded Theory approach, where theory was derived from the data collected.

**Results:**

Most participants were female (n = 78, 67%), and married (n = 110, 94%). All participant groups found  mentor mothers acceptable as women providing care to pregnant and postpartum women, and as WLHIV supporting other WLHIV. Mentor mothers were uniquely relatable as role models for WLHIV because they were women, living with HIV, and had achieved an HIV-negative status for their HIV-exposed infants. Mentor mothers were recognized as playing major roles in maternal health education, HIV treatment initiation, adherence, and retention, HIV prevention for male partners and infants, and couple HIV disclosure. Most WLHIV preferred to receive mentor mothers’ services at health facilities rather than at home, due to concerns about HIV-related stigma and discrimination through association with mentor mothers. Key mentor mother needs were identified as training, remuneration, and validation as lay health workers.

**Conclusions:**

Mentor mothers are an acceptable PMTCT intervention among stakeholders in North-Central Nigeria. However, stigma and discrimination for both mentor mothers and their clients remain a critical challenge, and mentor mother needs such as training, pay, and a sustainably supported niche in health systems require focused attention.

**Trial registration:**

Clinicaltrials.gov registration number (NCT 01936753), registered on September 3, 2013 (retrospectively registered).

**Supplementary Information:**

The online version contains supplementary material available at 10.1186/s12884-021-04002-1.

## Background

The risk of mother-to-child transmission of HIV (MTCT) can approach elimination if pregnant women are able to access quality, comprehensive prevention of MTCT (PMTCT) services. These services include antenatal care (ANC) that offers and facilitates maternal HIV testing early in pregnancy, prompt uptake of lifelong antiretroviral therapy for women who test positive, infant antiretroviral prophylaxis, early infant diagnosis, and support services to promote maternal and infant adherence to care and treatment [1–3].

In 2019, an estimated 150, 000 children acquired HIV *in utero*, at birth or while breastfeeding in 23 UNAIDS focus countries which had high numbers of children, adolescents and women living with HIV (WLHIV) [2]. These 23 focus countries include 21 African countries-of which Nigeria is one- which together account for 84% of the global number of pregnant WLHIV and 81% of children under 15 years who were living with HIV in 2019 [2].

As a high HIV-burden country, Nigeria has much ground to cover in its progress towards elimination of MTCT (eMTCT) among the nearly 100,000 HIV-exposed infants born annually [2]. The national PMTCT program started in 2001/2002 at six tertiary health facilities; by 2018, these services, supported mainly by the US President’s Emergency Plan for AIDS Relief (PEPFAR) and the Global Fund Against Tuberculosis, AIDS and Malaria, had expanded to nearly 6,500 health facilities across the country [4]. Despite the impressive scale-up of free-to-user HIV services, HIV treatment coverage among pregnant WLHIV in Nigeria was only 43% in 2019, compared to the 88% average among the UNAIDS focus countries [2]. With an early infant diagnosis coverage of only 27%, Nigeria ranked 16^th^ out of the 20 African eMTCT-focus countries in the same year [2]. The MTCT rate at six weeks post-delivery and from breastfeeding was 13% and 9% respectively, for an overall 22% MTCT rate in Nigeria [2]. These estimates are far above  the UNAIDS elimination targets of 2% and 5% MTCT rate among non-breastfed and breastfed infants, respectively [2]. Demand-side economic and social barriers (including funds for transportation, hidden user fees, and gender power dynamics) perpetuate low access to, and patronage of ANC and health facility delivery, two major platforms for PMTCT service delivery, contributing to unacceptably low HIV treatment coverage rates in spite of available services [5–8]. As a result, Nigeria alone accounted for approximately 15% of vertically-infected children living with HIV born globally in 2019 [2].

Several studies have shown that peer supporters and similar lay health workers who provide psychosocial and other support to pregnant and post-partum WLHIV enhance uptake of PMTCT services, which facilitate maternal viral suppression and infant HIV-free survival [9–12]. These lay peer supporters (also known as expert clients/patients/mothers or mentor mothers (MM)) counsel and guide other WLHIV through interactions at the health facility, home and/or other community locations [13, 14]. The pioneering South African mothers2mothers (m2m) PMTCT peer support program is now operating in 10 African countries; one in West Africa and the rest in East and southern Africa [14]. Similar models have been implemented in national PMTCT programs in many other African countries, often with significant funding from external donors [15–17].

While there is a growing body of literature on the impact of MM in PMTCT across sub-Saharan Africa [9–11, 13, 18], few studies have assessed the acceptability of these behavioral interventions among their target population (WLHIV) and other stakeholders [19]. These data are particularly lacking in the West and Central African region, where PMTCT achievements are lagging behind the rest of the continent [20].

The objective of this study, therefore, was to add to this growing body of knowledge specifically from West Africa, by assessing the acceptability of MM peer support as a PMTCT intervention among WLHIV and diverse groups of stakeholders in Nigeria. We further explore the opinions and experiences of participants regarding the wider PMTCT program, to identify barriers and facilitators to access and uptake of services, to guide MM program design and delivery.

## Methods

### Study design and setting

This qualitative formative study was conducted between December 2012 and April 2013, and was nested within the INSPIRE MoMent (Mother Mentor) study [21]. MoMent was a PMTCT  Implementation Research study  implemented in North-Central Nigeria between 2012 and 2017. With a prospective cohort design, the study evaluated the impact of a structured MM intervention on maternal retention and infant presentation for early HIV diagnosis [21–23]. MoMent was conducted at 20 rural and semi-rural primary health centres in the Federal Capital Territory and Nasarawa State in North-Central Nigeria. At the time of the study, rural and urban antenatal HIV prevalence in the Federal Capital Territory were 5.0% and 6.1%, respectively; and for Nasarawa state, 9.0% and 5.0%, compared to the national figures of 1.8% and 3.2%, respectively [24].

### Study population and recruitment procedures

We conducted focus group discussions (FGDs) and in-depth interviews (IDIs) with pregnant and non-pregnant women, including ANC attendees, MMs and m2m support group members. We also interviewed male partners, health workers, traditional birth attendants (TBAs), community leaders and PMTCT program implementers and policymakers [21]. FGDs allowed for an exploration of target group opinions and experiences. IDI participants were interviewed for their individual opinions in the context of their individual expertise and experiences. Participants were drawn from health facilities and surrounding communities within MoMent’s proposed study locations in rural Federal Capital Territory and Nasarawa State.

Eligible participants were recruited through purposive sampling and were ≥ 18 years old and living, working and/or accessing health services in the study communities. Health workers and community gatekeepers identified potential participants at health facilities or from the community, as PMTCT “users,” “facilitators” and “providers.” Interested participants gathered at agreed-upon interview locations, which were private spaces at health facilities or in the study community. Multilingual study staff explained the study and objectives to all participants in English or their preferred local language, and obtained individual written informed consent from each participant. Participants who could not read or write provided witnessed thumbprints.

### Data collection

Prior to each session, an interviewer administered survey was used to capture participants’ socio-demographic data, including age, marital status and religion. Two trained research staff facilitated each audio-recorded FGD and IDI using an interview guide; focus groups additionally had an observer to record non-verbal cues. At least one facilitator was multilingual (in English, pidgin English and/or Hausa, the dominant language in the study setting). The guides explored knowledge of/experiences with peer support, and its acceptability for pregnant women in general and for PMTCT in particular (see Additional Files 1, 2, 3, 4, 5 and 6). FGDs lasted 1.5 to 2 h and IDIs approximately 45 min.

### Data transcription and analysis

All FGDs and IDIs were transcribed verbatim; where necessary, they were translated into English from pidgin English or Hausa by the same staff who facilitated the interviews. Our theoretical approach to qualitative analysis was Grounded Theory, where we derived theory from the data, rather than applying preformed theory to the data [25, 26].

All transcripts were manually analyzed using the constant comparison method of Grounded Theory [26], in a thematic analysis approach [27]. The analysis team comprised a total of ten paired and trained research staff (including MAM, SE, NASA and LJC and the rest of the facilitator team). In initial coding, each transcript was hand-coded by one pair of analysts, who individually reviewed their assigned transcript line-by-line to identify recurring words and phrases as codes. Similar inductive codes were then collated into a list by each pair of analysts, according to identified patterns. Thereafter, in group analysis, these lists of codes were categorized into a coding tree, with parent (main) codes under which related subcategory codes were arranged [28]. This list was used to conduct a group iterative analysis, during which analysts’ interpretations were triangulated and themes elicited until thematic saturation was achieved and no more additional interviews were needed [29]. At this point, the listed codes did not identify any additional themes, and no more emerging codes or themes were derived from analysed transcripts. AO additionally analysed each transcript independently. We have provided supplementary information using the Consolidated Criteria for Reporting Qualitative Research (COREQ) checklist [30] (See Additional File 7).

## Results

We conducted nine FGDs and 31 IDIs with a total of 118 participants, including 20 pregnant women, 46 WLHIV (MMs and m2m support group members), 30 male partners, 17 health providers, and five community leaders (Table 1). Most participants were female (n = 78, 67%), and married (n = 110, 94%). Three recruited participants did not show up for interviews (one for an m2m FGD; two for MM FGDs); one MM was unable to participate due to a scheduling conflict, and information on the other two no-shows is not available.

**Table 1:** FGD and IDI participant characteristics

Participant Group (N groups)	Number of Participants	Female n (%)	Age Range (Years)	Education n (%)	Married n (%)	Religion n (%)
**Focus Group Discussions (9 groups, N = 87 participants)**						
**PMTCT Users (Women, including WLHIV)**						
ANC clients (2)	20	57 (100.0)	20–38	< Secondary: 18 (31.6) ≥ Secondary: 36 (63.1) No response: 3 (5.3)	54 (94.7)	Christian: 39 (68.4) Muslim: 18 (31.6)
m2m group members^a^ (2)	19					
Mentor Mothers^a^ (2)	18					
**PMTCT Facilitators**						
Male partners^b^ (3)	30	0 (0.0)	33–87	< Secondary: 12 (40.0) ≥ Secondary: 18 (60.0)	30 (100.0)	Christian: 12 (40.0) Muslim: 18 (60.0)
**In-Depth Interviews (N = 31)**						
**PMTCT Users (n = 9)**						
m2m group members^a^ Mentor Mothers^a^	4 5	9 (100.0)	21–50	< Secondary: 1 (11.1) ≥ Secondary: 8 (88.9)	9 (100.0)	Christian: 8 (88.9) Muslim: 1 (11.1)
**PMTCT Providers (n = 17)**						
Traditional birth attendants Health workers Govt. officials/policy-makers Program implementers	4 4 3 6	12 (70.6)	21–50	< Secondary: 2 (11.8) ≥ Secondary: 15 (88.2)	14 (82.4)	Christian: 14 (82.4) Muslim: 3 (17.6)
**PMTCT Facilitators (n = 5)**						
Community leaders^c^	5	0 (0.0)	21–51 +	< Secondary: 0 (0.0) ≥ Secondary: 4 (80.0) No response: 1 (20.0)	4 (80.0)	Christian: 1 (20.0) Muslim: 4 (80.0)
**Grand Total**	**118**	**78 (66.1)**	**20–87**	**< Secondary: 33 (28.0)** **≥ Secondary: 81 (68.6)** **No response: 4 (3.4)**	**111 (94.1)**	**Christian: 74 (62.7)** **Muslim: 44 (37.3)**

*FGD* focus group discussion, *IDI* in-depth interview, *PMTCT* prevention of mother-to-child transmission of HIV, *ANC* antenatal care, *m2m* mothers2mothers

^a^All women living with HIV (WLHIV)

^b^Male partners were not partners of WLHIV interviewed in the study. They were however recruited from the same communities, and some men were married to other WLHIV

^c^Includes 2 religious leaders, 1 teacher, 1 community development worker, 1 unemployed/volunteer

### Overall acceptability of mentor mothers among stakeholders

There was consensus among all categories of respondents that MMs were acceptable, as women providing peer support to pregnant women, and as WLHIV who support other WLHIV.

#### PMTCT users: pregnant and non-pregnant women living and not living with HIV

Peer support was acceptable to women in general, and especially first-time mothers, because they could receive psychosocial support and experiential advice on pregnancy and infant care-related issues.

Like we that have not gotten pregnant before; it might be good if I have somebody like that, who will talk to me and make me feel relaxed. (Primigravid woman, FGD ANC 1)

For WLHIV, MMs were healthy and had HIV-negative children, and were therefore role models for optimal maternal health and having an HIV-free infant.

It would encourage me [to have an MM]. She would be encouraging me to do the same thing that she did during her pregnancy. (WLHIV 1, IDI)

Without them (MMs), we will not know how to take our drugs or take care of our babies. (m2m 1, IDI)

#### PMTCT providers: Healthcare workers, including traditional birth attendants, program implementers and policy-makers

Health workers and other providers were accepting of peer support in PMTCT because of the positive impact of MMs on WLHIV outcomes – results that sometimes, health workers were unable to achieve.

Most of the time when a woman is [HIV-] positive, no matter how the health worker advises her, she doesn’t accept it like from the mentor mothers, because the mentor mother will tell them how they were successful through PMTCT. So mentor mothers are very, very important. (State Ministry of Health staff, IDI)

They [MMs] are useful because they encourage women to come to the hospital. (TBA 1, IDI)

Some [pregnant WLHIV] will say [to health workers], “Ah Aunty, you don't understand what I’m passing through.’ But if the mentor mothers, those that have passed through the same thing, talk to her, they get relief. So they [MMs] help us. (Health worker 1, IDI)

They will allay the fear of the newly diagnosed positive patient; knowing that she has been in your shoes, she has passed through it, and gave birth to children [who] are HIV-negative. It will really serve as a motivation for the mothers. (Program Implementer 1, IDI)

#### PMTCT facilitators: male partners and community leaders

Male partners were favourably disposed towards peer support for pregnant women, regardless of the woman’s HIV status.

The services these ladies [MMs] are going to do is similar to the ones our mothers would have been doing for our wives if our wives were to be living with them. (Male partner, FGD1)

They [MMs and WLHIV] will both understand themselves from experience, being they are both HIV-positive. They will communicate better. (Male partner, FGD1)

Based on MMs' experiences, male partners for the most part encouraged and supported their HIV-positive partners to work as MMs, regardless of the man’s own HIV status. However, stigma was a concern:

The husband has no problem; he only told her [MM] that she should ensure that she is accepted where she goes for the visit. And that she should not enter any house where they don’t accept her. (MM, FGD2)

Other male partners, however, did not approve of their partners’ work as MMs; some withdrew financial and material support.

Since she started working as a mentor mother, the husband has stopped supporting her with little things that she is in need of, like soap and household items. And during their meetings, other women usually complain that their husbands no longer provide for them. (MM, FGD2)

Community leaders also expressed the need for mentor mothers and were in support of them working in their communities:

It is not somebody new that is coming to the community to tell them [women and their families] “Do this, do this” because if somebody is coming from outside, somebody that they don't know, they may not be convinced… so the best thing is to use such, you know; mentors, such people from the community to talk to the community people. (Community leader 1)

(Mentor mothers will be helpful) because they [MM] have been trained and they are participating already [in PMTCT] and because they volunteer to participate. The women understand them and they are used to them and the mentor mothers have gained knowledge as a result of the training they received and they can use their knowledge to create more awareness. (Community leader 2)

### Role of mentor mothers

WLHIV who were MMs and m2m group members spoke from their personal experiences of what MM roles were and/or should be.

#### Positive role model and confidante

As WLHIV who have utilised PMTCT services with successful outcomes, MMs are uniquely positioned to model positive HIV prevention, treatment uptake, adherence and retention behaviors for other WLHIV to emulate.

When we do our post-test counselling, we usually tell them [WLHIV] that we are in the same shoes as they are. They can’t believe it. When we tell them that we are also positive, they will say that it is a lie. (MM, FGD2)

Three main characteristics make MMs uniquely relatable and credible to their WLHIV clients. First, they are women, and thus understand the gender-related challenges of life, health, and healthcare in the community. Second, MMs are also living with HIV and therefore share the same lived experiences. Third, MMs have successfully navigated the PMTCT cascade resulting in an HIV-free infant, so they can provide guidance to other WLHIV from these personal experiences. Ultimately, MMs play roles for WLHIV that cannot be played by health workers, male partners or other women not living with HIV.

When we came for the [m2m] meeting it was all women, and two people were here to tell us many things about this mother-to-child stuff. So you feel more free discussing with your fellow woman who is positive within the circle. (m2m member, FGD1)

They counsel you as direct experienced persons…telling you what they passed through. It worked for them so it will work for you if you copy it. (WLHIV 2, IDI)

As a mentor mother, you can talk the way they [WLHIV] will understand. But as a nurse, if you tell them something, they will be like, “don’t mind that nurse, she doesn’t know what I’m passing through”. (WLHIV 3, IDI)

#### Emotional and psychosocial support

The first resource a newly HIV-diagnosed pregnant woman often encounters is an MM, during one-on-one post-test counselling [13]. This initial encounter provides critical emotional and psychosocial support to help the client understand and deal with the ensuing anxiety, fears and concerns following a positive HIV diagnosis.

Most of them [newly-diagnosed pregnant women] respond by worry. So we usually calm them down and tell them that we have been in this situation and most of them calm down; they cooperate with us and accept the information given to them, including how they can get the drugs so that eventually their children will not be able to contract HIV. (MM, FGD2)

MMs are also instrumental in forming m2m support groups for WLHIV. They facilitate regular group meetings during which they provide health education, psychosocial support, and HIV treatment literacy.

During health education, they [MMs] educate the mothers. (Health worker 2, IDI)

After the post-test counselling we enrol the [HIV-positive] person into the mother-to-mother support group. So whenever they are having meetings, we call them. (Health worker 3, IDI)

#### HIV prevention for women and their male partners

MMs play a key role in HIV prevention through pre-and post-test counselling for pregnant women during ANC clinics. Prevention messages include breastfeeding and safe infant feeding options to prevent infant HIV infection. For women who test HIV-positive, MMs also encourage and assist with male partner HIV testing and couples’ HIV status disclosure.

When the results come out, she [nurse] hands them over to me. I know how to tell a positive woman to bring her husband. I will tell you to tell the man that they want to see him in antenatal [clinic]. Then I will know strategies to use, and those strategies have been working. This month we counselled twelve [men], and out of those twelve, four were positive, and I know how I’ve tackled those that come out positive. (MM FGD1)

#### Support for women throughout the PMTCT cascade

Women who test positive are encouraged by MMs to initiate HIV treatment during post-test counselling. MMs also follow up on WLHIV through phone calls, text messaging and/or home visits to encourage adherence, keeping clinic appointments and attending support group meetings, which ultimately facilitate retention and sustained viral suppression.

At times they [newly-diagnosed WLHIV] will not listen to us. But when they [MMs] tell them, “See, I have gone through it and I’m breastfeeding my baby and the baby is HIV-negative,” then most of them that refused will start taking the drugs. (TBA 2, IDI)

[During home visits], we remind them about their drug, discuss their well-being and then clinic attendance if they forgot. (MM, FGD2)

[MMs] are very effective in tracking [clients who missed appointments]. (Program implementer 2, IDI)

Mentor mothers follow them up so that they bring them back so that they especially receive nevirapine for the babies. (Program implementer 3, IDI)

#### Mentor mother recruitment

The m2m support group is a natural and convenient platform for recruiting MMs. Health workers often select MMs based on their demonstrated leadership and commitment in m2m support groups and consistent clinic attendance and treatment adherence.

The matron in charge saw me coming all the time to collect my drugs. So she asked me if I could continue mentoring other mothers and bringing people to the hospital for testing. (MM FGD2)

The matron of the hospital talked to me. I told her I have a diploma. She said, ‘Can’t you come and give health talks to these women since you can talk? That is how I started coming out and giving health talks to those women. (MM, FGD1)

Members of support groups may also indicate their interest to work as MMs.

When we started the support group, she [nurse] announced that we need mentor mothers, and any mother that passed through PMTCT, who is willing to do it, should come and join. So I said I will join because I want to share my experience with other women. (MM FGD1)

While MMs were regarded as a welcome community resource, there were reservations about recruiting MMs to work in their own community, due to stigma.

Because [MMs] live with them in the community, [WLHIV] won’t have any inferiority complex when they are talking to [MMs]. (Community leader 1, IDI)

Women will not agree to be mentor mothers in their community because that would be the same as declaring that they are HIV-positive. (TBA 2, IDI)

Other personal qualities of potential MMs include being approachable, possessing good interpersonal and leadership skills, resilience, and acceptance of their own HIV-positive status as a demonstration of overcoming (self)-stigma.

From the support group members, when they see a very strong somebody with potential of being a leader, they can recruit the person into the mentor mother program. (MM FGD1)

The way I took the news, maybe [the nurse] was thinking I will break down and start crying, but I took it in good faith. Then, she talked to me about being a mentor mother. (MM FGD1)

MMs were expected to be willing to share their personal PMTCT experiences with other women, and disclose their HIV status to their clients.

From m2m [support groups], we choose those that are willing to share their ideas, challenges and experience to be a role model and help use themselves as a testimony. (MM FGD1)

Self-disclosure was particularly important, as some clients had reported that their MMs did not directly disclose their HIV status to them, if at all.

She [MM] did not disclose her status to me and I did not know she was hiding it. It was the day we came for a [m2m] meeting here, after about a year plus, that I came to know. (M2M FGD1)

### Facility versus community-based peer support

WLHIV overwhelmingly preferred receiving MM support at facilities as opposed to home visits, because of the fear of being “outed” as living with HIV. While MMs themselves were not opposed to conducting home visits, they reported that the majority of their clients preferred facility-based support. This was due to the fear of association with MMs, who were known in the community to be HIV-positive.

Out of 100, I think it is only 25% that allow us to go to their house. 75% don’t want us to come because maybe they’re having partners [co-wives] at home and the partners maybe have not been tested, and maybe the man too, is a kind of a man that, when you tell him something, he will be talking to everyone about it. (MM FGD2)

Women who did not want home visits preferred mentoring by telephone.

Honestly, when they know that you are “this” [HIV-positive], they don’t want you to come to their house. So normally we contact them with phone. (MM, FGD 2)

A few WLHIV, however, preferred home visits because they did not want to be seen in the clinic, as frequent clinic visits could increase the risk of revealing their HIV-positive status.

Some will feel that since she has HIV, people will know, and she doesn’t want to be coming [to the clinic] so that people will see her. She will prefer to call somebody to come home and check her. (ANC FGD2)

The underlying reason for women choosing to interact with MMs at home or at the facility appeared to be perceived stigma, and fear of lack of privacy and confidentiality. The risk of being gossiped about by neighbours also varied with place of residence, being perceived more common in rural areas and places where people lived in proximity.

It depends on the environment you live in. They have neighbours all round. And you know some women are fond of gossiping. So if they get to know about something like that, the way they will talk about the person….So I don’t really think it’s ok [for home visits]. (ANC FGD1)

### Duration of mentor mother support

Participants suggested that MMs provide support to clients throughout pregnancy, delivery, and post-partum.

Let me say: throughout my pregnancy and after my delivery—maybe one or two months, they [MM] can stay with me. (ANC FGD1)

She should be supported for six months after delivery. (Male partner FGD1)

Some women felt that the duration of support should be inversely proportional to parity, so that primigravid or primiparous women would receive support for a longer period.

It depends on which pregnancy you are carrying [ie parity]. (ANC FGD1)

### Program and material support for mentor mothers

#### Training

Specific and tailored training for MM on HIV and communication skills were considered necessary to enable them to function properly.

We the mentor mothers, if you [PMTCT program implementers] will set a kind of training for us, we will so much appreciate it. (MM FGD2)

We also need training. We have to know how to talk to [the] husbands. (MM FGD1)

#### Financial support

Adequate funding-at least to cover transportation costs for home visits, support group meetings, phone calls and text messages to clients-were needed as fair compensation and to incentivize MMs’ performance.

Mentor mothers [should] be provided with a token, because they are the people who go to the villages. (State Ministry of Health 1, IDI)

If [an MM] can be employed, she knows that she is being paid fully for the work she is doing. She will not joke with it. (State Ministry of Health 2, IDI)

We have the mentor mothers who track our lost-to follow-up within the community. We usually give them an incentive monthly, and money for recharge cards. (Program Implementer 2, IDI)

#### Identification cards and attire

Some MMs mentioned the need for identification cards and/or special attire for work.

Our greatest challenge in going into the community is the issue of ID cards, because people want to know who exactly we are before going into their compound or visiting them at home. (MM, FGD1)

However, others felt these would increase HIV stigma and discrimination in the community.

How can you wear an ID with HIV on it and visit somebody? The people within that compound will know that the particular person that you came to visit has HIV. (MM, FGD1)

Similarly, some respondents stressed that attire that easily identified the MM as a person living with HIV or working with people living with HIV should also be avoided.

Even this our shirt, some people will say if you are coming to my house don’t wear anything that will display HIV. (MM FGD1)

**Fig. 1:**
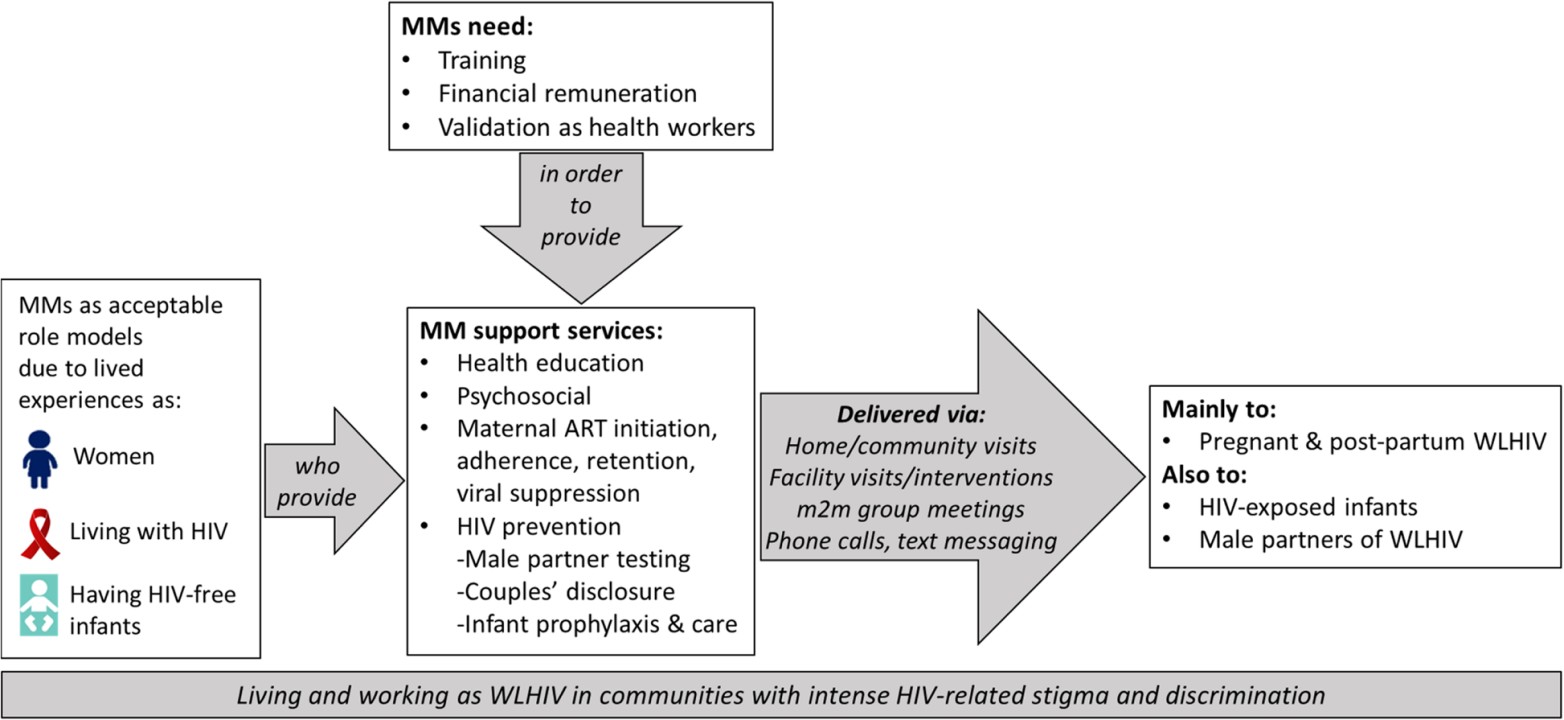
Emerging themes on mentor mother peer support to women living with HIV

Figure 1 presents the above emerging themes and their interrelationships as derived from our thematic analysis.

### Application of formative research findings to study design

Amenable findings and lessons learned were applied to the prospective cohort study design. These focused largely on the design and delivery of the MM intervention, and included changes in MM selection requirements, locations for client interactions, attire, certification of MM training, and modalities for paying stipends (Table 2).

**Table 2:** Changes made in MoMent study design based on formative research findings

	**Original Study Design**	**Formative Findings**	**Adjusted Study Design**
1	MMs to interact with WLHIV clients only at facility and at client homes	Due to stigma, WLHIV and their families may not want to receive home visits from MMs, who lived in the same communities and were often known to be HIV-positive	MMs to provide services at facility and at a variety of community locations including home, religious centers, markets and agreed-upon locations. Documented phone calls also count toward MM interactions
2	Branded attire for MMs to wear at facility and/or in the community	Even if MMs wanted to wear identifying attire, clients and their families preferred them not to, due to intense community stigma	MMs are not to wear branded or any other distinguishing attire for their work activities
3	MMs to be trained with study curriculum without receiving documentation of training	MMs took their jobs very seriously and were motivated as both patients and service providers in the PMTCT program	Each study-trained MM provided a formal certificate upon completion of training to foster belonging in the PMTCT program and recognition from health workers and clients
4	MMs were required to be able to read and write English at least at 5^th^ grade level to participate, due to documentation needs	Ability to communicate effectively with WLHIV clients in their first language was more important for MMs’ impact than being able to read and write English	MM training was conducted in both English and Hausa, the dominant local language. English literacy was made a desirable but not required MM skill. MMs lacking this skill were assisted in documentation by English-literate MM supervisors
5	MMs to be paid through the health facility’s PMTCT operational procedures, which could experience administrative delays	Fair and consistent compensation was critical to MM motivation and performance	MM stipends (~ $50 monthly) paid directly to them via personal bank accounts or through local chapters of peer WLHIV associations

*WLHIV *women living with HIV, *MM* mentor mother, *PMTCT* prevention of mother-to-child transmission of HIV

## Discussion

Our findings indicate that MMs were acceptable to WLHIV and other stakeholders in our study setting. Similar findings have been reported from Kenya [19], and among low-income women in the United States [31], and England [32]. A recurring phrase in our study was that MMs ‘were in the same shoes’ as their WLHIV clients. This similarity of lived experiences and personal demonstration of successful PMTCT outcomes make MMs relatable, credible role models [31–35].

MMs facilitate key services along every step of the PMTCT cascade [13, 33, 34, 36–38]. Similar to prior studies, we found that keeping exposed infants HIV-free was a particularly strong motivator for WLHIV to access and remain in PMTCT care [32, 33, 39, 40]. MMs project relatability and empathy to further motivate WLHIV to access and stay retained in care, which formal health workers are sometimes unable to do. We have previously documented the narratives of WLHIV (including MMs) regarding negative attitudes of health workers in Nigeria [5, 41], and McLeish (2016) and Shroufi (2013) described empathy from MMs that WLHIV felt health workers did not possess or demonstrate [32, 34]. As with other African studies, our study respondents preferred MM support to last from pregnancy through several months post-partum [36], with lower-parity women receiving longer-term support than more experienced mothers.

Despite the overall acceptance of MM peer support, stakeholders echoed concerns about privacy and confidentiality during home or clinic visits from MMs, due to fear of inadvertent HIV-status disclosure and the ensuing stigma and discrimination. This has also been reported from Kenya and Malawi [33, 36, 42]. Such concerns factor into WLHIVs’ preferences for home, phone, or clinic-based support, and whether recruited MMs should work in the communities where they live [19]. As also reported from Kenya, clients also did not want MM to wear ‘HIV-identifying’ attire or identification cards [19, 36].

Similar to other studies, we identified essential MM qualities such as good communication skills, empathy, friendliness and willingness to share their own PMTCT and lived HIV experiences. The finding that some MMs did not disclose their status to their clients was unexpected and concerning. This is contrary to the assumption that all MMs had overcome (self)-stigma and were therefore willing and unafraid to disclose their HIV status. We have published detailed findings on MM non-disclosure from the same Nigerian study setting [43].

Another MM requirement was an explicitly expressed willingness to play this role. Simply possessing other desirable qualities was not enough to recruit an MM: she must also be enthusiastic and motivated to provide peer support. While this may seem obvious, implicit in this is the commitment to positive maternal, infant and family PMTCT outcomes and openness in sharing personal experiences, including a positive HIV status.

Public disclosure by MMs in community settings is a means of normalizing living with HIV – as these women reflect the overcoming of (self)-stigma and living healthy and fulfilled lives. Creating community awareness about the availability and impact of PMTCT interventions also contributes to removing the stigma around HIV. The overwhelming concern for privacy and confidentiality stemming from lived and observed stigma and discrimination echoed through discussions with all participant groups. The avoidance and/or mitigation of stigma would therefore impact on MM recruitment and all aspects of MM activities along the PMTCT cascade.

The recommendation for adequate MM compensation illustrates MM acceptability, and could promote program sustainability through improved lay worker satisfaction and performance [41, 44–48].

### Study strengths and limitations

Our study strengths include the diversity of recruited stakeholders: WLHIV with and without prior MM experience, pregnant and non-pregnant women who had neither PMTCT nor MM experience, formal (health worker) and informal (TBA) maternal care providers, program implementers and policy makers, male partners, and community leaders. However, our findings are from North-Central Nigeria, and thus may not be generalizable to other Nigerian or African settings. The MoMent study did not focus on adolescent respondents < 18 years, whose experiences may be different—both as PMTCT providers/peer supporters and PMTCT users. Thus, our study findings may not be applicable to this group. Lastly, it was evident that some participants in our study setting were already familiar with the concept of MMs in PMTCT programs. It is possible that the level of acceptability of this peer support intervention may have been different if there were little to no prior exposure to the concept among our participants.

## Conclusions

This qualitative study provides insight regarding the local context for acceptability and application of PMTCT peer support in Nigeria. Findings informed the redesign of an MM intervention to better suit the needs and circumstances of WLHIV and communities in North-Central Nigeria, in order to facilitate improved health outcomes. Per our findings, lay maternal peer support plays a critical role in PMTCT and is acceptable to WLHIV and other stakeholders. It is important, however, to address pervasive issues around privacy, confidentiality, stigma and discrimination for both MMs and their clients. As highlighted by UNAIDS, inequalities, stigma and discrimination undermine efforts towards HIV epidemic control by contributing to increased vulnerability, limited access to care and treatment, HIV acquisition (including MTCT), and death from AIDS-related illnesses [49]. Failing to address these issues will continue to limit utilization of scaled-up, available PMTCT services. Lessons learned from implementation and additional research in West and Central Africa should further inform MM programs to support countries in addressing PMTCT gaps in this region. Finally, considerations are needed to create and sustain a niche for MMs in PMTCT and in general maternal-child health.

## Supplementary Information

**Additional file 1.** FGD guide for PMTCT users: pregnant women at antenatal care clinics.

**Additional file 2.** FGD guide for PMTCT users: women living with HIV (m2m and MMs) [43].

**Additional file 3.** FGD guide for PMTCT facilitators: male partners [8].

**Additional file 4.** IDI guide for PMTCT users.

**Additional file 5.** IDI guide for PMTCT providers (formal health workers, traditional birth attendants, government officials/policy-makers, program implementers).

**Additional file 6.** IDI questionnaire for PMTCT facilitators: community leaders.

**Additional file 7.** COREQ checklist.

## Data Availability

All data generated or analyzed during this study are included in this published article (and its supplementary information files).

## Abbreviations

ANC: Antenatal care
eMTCT: Elimination of mother-to-child transmission of HIV
FGD: Focus group discussion
HIV: Human immunodeficiency virus
IDI: In-depth interview
MM: Mentor mother
m2m: Mothers2mothers
MTCT: Mother-to-child transmission of HIV
PMTCT: Prevention of mother-to-child transmission of HIV
TBA: Traditional birth attendant
UNAIDS: Joint United Nations Program on HIV/AIDS
WHO: World Health Organization
WLHIV: Women living with HIV
