# Supplementary material for: Acceptability of mentor mother peer support for women living with HIV in North-Central Nigeria: a qualitative study
Source: BMC Pregnancy Childbirth. 2021 Aug 7;21:545. doi: 10.1186/s12884-021-04002-1 (PMC8349095; doi:10.1186/s12884-021-04002-1)
Supplement: Supplementary file 1 — Additional file 1. FGD guide for PMTCT users: pregnant women at antenatal care clinics. [file 12884_2021_4002_MOESM1_ESM.pdf]

**MoMent ANC FGD QUESTIONS**

If there are no further questions, we would like to begin the discussion. **(Begin recording, state date, type of FGD, and venue)**

# I. Main Discussion

## **The initial question** (10 minutes):

Let's talk about pregnant women and how we take care of them in the clinic.

1. What do you think about the services pregnant women receive in the clinics in this area? Do pregnant women visit the clinics a lot? Why or why not?
   Probe: Fear of/apprehension with going to the community clinic was mentioned a lot. Tell me a bit more about that.

2. What kinds of services are you getting/did you get? ; Do you think it is enough? What about the quality of services? Do you think it is better to come here than to deliver at home?
   Probe: if some of the group members talk about delivery at home ask?
   Tell us about how/why you would deliver at home/with the traditional birth attendant?

3. What about if the pregnant woman has HIV? Do you know anything about the services a woman can receive from a clinic if she has HIV? Probe: What do you think about that?

## **ANC SERVICES/ DEMAND (for all groups)** (10 minutes)

The next part of the focus group looks at how close the clinics are, and the types of ANC services we provide.

1. First, tell us a bit about how you travel to the clinics and any challenges you face in getting here. Probe: Are the clinics too far from where you live or hard to get to?

2. Next, tell us about how you felt you were treated when you came into the clinic to get medical care. What typically happens- are you seen right away or do you have to wait long? Probe: Why is that the case? Probe: Do you feel that the staff treat you with respect and listen to you?

4. Are the staff usually ready to attend to you?
   Probe; If not what do you think we should do to improve this?

5. If you have to get any blood tests, do you have any problems with them obtaining the blood? Do you get your results on time?

6. Do you have any special requests- such as needing to be seen only by a woman? Probe: What other things would make you feel more comfortable in the clinic?

7. How has the staff responded to these needs? Probe: Overall have you found the staff to be welcoming?

8. Have you found any problems with local politicians or chiefs (slowness or refusal to respond to problems with health clinics) that have made it difficult for you to use the clinics? Probe: If there are problems, do you think the government can be blamed for some of the problems with the clinics? Why do you think the government has to be blamed for this? Probe: What suggestions do you have for solving the problems?

**Discussion of Acceptability and efficacy of Mentor Mother (Mother Helper) programs (10 min)**
The next part of the focus group looks at the types of support pregnant mothers have received while using some of our clinics. For the next 15 minutes, we would like to talk about Mentor Mother (Mother Helper) support. These women have been pregnant before; they have used and know the clinic services very well.

1. First, how do you feel about having a helper to talk to you, as a pregnant woman, about services that will be useful to mothers and their babies?
Clarify: the services include how to feed the baby and prevent infections, remind you to take any medicines you may have, how to take care of yourself after having the baby, how to use the clinic services, and to get immunizations and how to get to the big hospital if you had a serious problem.

2. If you needed a helper, would you allow them to visit you at home?
Probe- Did you or are you open to have a helper support you? If so what are the types of things you would like for them to help you with?
Probe: How can we use helpers to reach more mothers like you?

3. If you would accept a helper assigned to you from the clinic, how long would you like them to help you? When the baby is 1 week, 1 month, 3 months, 6 months, even up to 1 or 2 years?

4. Do you think women with HIV can benefit from the services and the helper we just talked about as well? Probe: Why, or why not?

**Discussion of Communication – issues related to language, being talked with and heard** (10 minutes). We are now going to talk about whether you think the clinic workers are talking to you and listening to you.

1. Tell us what happens when our doctors, nurses and other staff members talk to you about being pregnant? Do they teach you anything about pregnancy and how to be healthy?
2. Do you think the clinic workers talk to pregnant mothers who are HIV positive as well? Probe: What do you think they tell them?
3. Do you feel they understand what you were trying to tell them?
   Probes: Did they speak the same language? Did you feel that you were being ignored or when you speak?

**Discussion of Treatment – use of medical practices (formal and informal practices)** (5 minutes):
The next discussion is focused on the clinic and home medicines you use to take care of yourself.
1. Describe the things you do (medicines you take, home remedies and so on) or healers you visit to take care of yourself?
2. What types of services like medicine, nutritional/food support do you receive at the clinic?
3. Tell us about if you, as a woman or mother, ask others in your community for help in caring for yourself when you are sick or have other problems.

Probes: What types of people do pregnant women or new mothers go to when they need help with themselves or the baby, and why?
4. What are the things they do to help you/the mothers with these problems? -
5.

**Discussion of** Stress/**Stigma – an exploration of the issues of stress** (10 minutes):

The next discussion is focused on the perceptions of stress and stigma related to pregnant women -.
1. Tell us about the types of stress pregnant women have to deal with in pregnancy. What about stress with a new baby?
2. Do pregnant women or new mothers discuss/share problems with anyone in their community, outside of their family?
3. What about moms that have HIV/AIDS- what kinds of stress and problems do you think they face?
   Probes. How often do you feel stressed? Do other people your stress?

# II. Closing (10 min)

We would like to finish our discussion by asking if there are any suggestions you have for us or anything you would like to say in closing. (comment: allow time for general discussion).

We would like to thank you for spending time with us, we appreciate all that you told us and look forward to using this information to help improve the services we provide. (Comment**:** Issue their compensation if immediately available or explain the payment process if not.
