# Supplementary material for: Acceptability of mentor mother peer support for women living with HIV in North-Central Nigeria: a qualitative study
Source: BMC Pregnancy Childbirth. 2021 Aug 7;21:545. doi: 10.1186/s12884-021-04002-1 (PMC8349095; doi:10.1186/s12884-021-04002-1)
Supplement: Supplementary file 3 — Additional file 3. FGD guide for PMTCT facilitators: male partners [8]. [file 12884_2021_4002_MOESM3_ESM.pdf]

MoMent Study Questionnaire Guide for Male Partners

Married men; HIV status or that of wives irrelevant to participation in FGD.

If there are no further questions, we would like to begin the discussion. (Begin recording)

MAIN DISCUSSION

Icebreaker

We know that as a man, you are considered a leader in your home. Do any of you have leadership positions in the community? eg Imam, mallam, pastor, chief, traditional advisor, medicine man, etc.

The initial question (20 minutes): **Health-Seeking Behavior for self and family**

Self *(Ask about religious, traditional or other reasons for any relevant answers)*

1. Let's talk about where you go for help if you are sick.
   Probe: Do you use private or government clinics/hospitals?
   Probe: Are you taking any medicines for your health right now, like for diabetes or high BP? Did you get these medicines from a clinic; do you use traditional medicines, or both? Do you think one is better than the other?
   Probe: In what situations would you use clinic medicine versus traditional medicine?

Children

2. Are any of you married? Do you have children? Where do you send the children when they are sick?
   Probe: Do you give them traditional medicine or send them to the clinic, or both? Tell us more about that.

Wives:

Some/all of you said you were married. How many of you have more than one wife?

3. Where do you send your wives, or where do your wives go, when they are sick?

Women, Maternal Healthcare Services and Knowledge

Ask about religious, traditional or other reasons for any relevant answers

4. What about when your wives are pregnant?
   Probe: Do they go to the clinic, or are they taken care of at home? Do they deliver in the clinic, or at home?
   Probe: Do some of your wives deliver at home, and others at the clinic? Please explain why.
   Probe: What are the good things about sending your wives to clinic when they are pregnant? What are the bad things?
   Probe: Do you have to give permission to your wife before she can attend clinic? Are you okay with her attending clinic but delivering elsewhere?
   Probe: would you prefer your wife to deliver in the clinic or at home? Why or why not?

5. What services do you think a pregnant woman receives or should receive when she goes to the clinic?
   Probe: What kind of advice, tests, drugs, do you think she should be getting?
   Have your wives who have gone to clinic received these services?

Probe: How many times do you think a woman visits or should visit the clinic before delivering? How many times did your own wives visit the clinic before they delivered?

6. Have you ever attended ANC with your wife when she was pregnant?
   Probe: why did you go with her? Was it because she couldn't travel alone, or because you wanted to accompany her to the appointment?
   Probe: If not, why did you not go with her? Does anyone else usually go with her?

7. Tell us a bit about any challenges you/your wife may have in attending clinic, if you have both attended clinic before.
   Probe: Are the services too far from where you live or hard to get to?
   Probe: what about the road conditions?
   Probe: What about cost of the transportation?
   Probe: What about the kind of treatment you get/she gets in clinic? What usually happens- is she seen right away or do she have to wait long?
   Probe: Did the staff treat her with respect and patience? Did they listen to her problems? Did she get proper care?

8. ***(Poll the room)*** Does it make a difference for you whether a man or woman attends to your wives, especially during delivery? Are there any religious or traditional/cultural reasons why?
   How has the staff responded to these needs?
   Probe: What do you think about traditional birth attendants as against the hospital facilities for caring for women?
   Probe: For those of you feel that Traditional Birth Attendant services are better or worse, what are the reasons why?
   Probe: How can we improve the clinic services to make them acceptable to you and your wife?

General HIV knowledge and education among men/male partners

9. I am sure most of you have heard about HIV. Tell us what you know about HIV disease.
   Probe: Have you heard any radio announcements or seen any billboards/signs about HIV?
   Probe: Where do you think HIV comes from? How does one person pass it to another?

10. We talked a little bit about what you know about HIV. Where did you get most of the HIV knowledge that you have?
    Probe: was it doctors, nurses, radio, billboards, newspapers, friends, etc?

11. Do you feel that HIV information/education is directed more to women or men, or equally for both?
    Probe: What about unmarried vs married people?

12. What are the ways in which you think HIV information can be directed more towards men?
    Probe: What would you like to know more about? How would you like to get this information?

Knowledge and Attitudes re: Pregnant women living with HIV

13. Did you know that every pregnant woman who attends the ANC will get a free HIV test? Why do you think she has to get this test? (Best answer is: to know her status so that she can be taken care of with drugs, as well as to prevent the infection from passing to the baby. Please clearly state this if no one comes up with this statement).
14. Did you know that if a woman with HIV did not know she was positive and she gets pregnant, she can pass the HIV to her baby?
15. Do you think there are /there should be any differences in the care HIV+ women get from clinics?
    Probe: What about the kinds of services they get? What about how the staff treat them?

16. Do you think a woman with HIV should deliver at home (vs clinic)?
    Probe: Why or why not?

Discussion of Stigma – an exploration of the issues of stress and stigma relating to living with HIV/AIDS (15 minutes):

The next discussion is focused on the perceptions of stress and stigma (bad treatment).

17. Tell us about the things that make you have stress, problems or thinking.
    Probes. How often do you feel stressed/tension?

18. Now let's talk about people with HIV. What do you think are the types of problems that people with HIV/AIDS have to suffer?
    Probe: What do you think about that? Do you know anyone with HIV who has been treated badly?

Acceptability of Mentor Mothers among male partners

The next discussion focuses on help that pregnant women may need.

19. What kind of help do you think would be useful for a pregnant woman?
    Probe: How long do you think she will need that help?
    Probe: Who do you think would be the best person/people to help her?

20. Do you think pregnant women have stress/tension? How best can we help them?

21. Sometimes clinics send an experienced and trained mother to help a pregnant woman to manage her pregnancy, teach her how to take care of her baby, and help to reduce her tension/stress. What do you think about that?
    Probe: ***(Poll the room)*** Would you accept that for your wife, if she said she wanted the help?
    Probe: ***(Poll the room)*** Would you be okay with it if this helper were to visit your wife at home?

22. What about for HIV-positive women? Do you think that such help would be useful for them?
    Probe: Why or why not?

System, Political and Leadership Roles in ANC attendance and quality of care

23. What do you expect the Nigerian government or Ministry of Health to do, to improve clinic services and attendance for pregnant women?

<u>Probe:</u> What about electricity, roads, clinic staff, (number, their training, their pay), equipment, drugs, opening hours, community engagement and awareness?

24. What do you expect your local government chairman and village chief to do, to improve clinic services and attendance for pregnant women?

Closing (10 min)
We would like to finish our discussion by asking if there are any suggestions you have for us or anything you would like to say in closing. (Allow time for general discussion).

We would like to thank you for spending time with us, we appreciate all that you told us and look forward to using this information to improve the health services that are provided to your community, especially for women and children.

END.
