# Supplementary material for: Acceptability of mentor mother peer support for women living with HIV in North-Central Nigeria: a qualitative study
Source: BMC Pregnancy Childbirth. 2021 Aug 7;21:545. doi: 10.1186/s12884-021-04002-1 (PMC8349095; doi:10.1186/s12884-021-04002-1)
Supplement: Supplementary file 4 — Additional file 4. IDI guide for PMTCT users. [file 12884_2021_4002_MOESM4_ESM.pdf]

INSPIRE MoMent PMTCT Study

Users' Group: In-depth Interview

Interviewer's Name:

Date: Day/Month/Year

Participant specific group: ☐ **Superusers (MM/M2M)**

Introduction: *The purpose of this key informant interview is to gather data from the informant about:*

- *Challenges in PMTCT implementation, access, service delivery and retention*
- *Suggestions for how these challenges may be solved*
- *Acceptability of a Mentor Mother program in community*
- *Suggestions for how a Mentor Mother program may best be implemented*

The information we collect will only be reported together with information collected from other interviews. The names of informants will not be connected to the information collected.

I. Background

Section Introduction: *The purpose of the first section of the interview is to understand your experience as a PMTCT user/or how you missed PMTCT services.*

Are you a Mentor Mother or M2M support group member?

When did you <u>first</u> hear about PMTCT? (Month and year) How did you hear about it?

What was your reaction/feeling when you first heard about PMTCT? Do you feel the same way now?

Where did you first access PMTCT services? (Primary healthcare facility, General Hospital, private?)

II. Assessment of PMTCT Implementation

Section Introduction: *The purpose of the next section of the interview is to understand how you feel about the way we are providing PMTCT to the community.*

a. Please describe your best experience with PMTCT (or the best information you heard about PMTCT-if you have never used it).
b. Now please describe your worst experience with PMTCT services.
c. Please describe, in your own understanding, how you think PMTCT services should be provided to the community. *Probe: what do you think we are doing well? What do you think we are not doing well? How can we improve?*

d. Why do you think that some people with HIV miss their appointments and do not take their drugs properly? *Probe: personal, provider, site, community factors*

e. What do you think the clinics should do if someone is not taking their drugs or misses their

	appointments? *Probe: do you think we should call them or send someone to their home?*
f.	**For those who used PMTCT**: Do you think the clinic took care of you very well during PMTCT? What happened when you missed appointments or tests? Did they ask you about your drugs?
g.	**For those who used PMTCT:** What did they tell you about the baby? Probe: did they tell you about collecting drugs for the baby as soon as they were born, test at 6 weeks? Did you get all the drugs and tests on time? Why or why not?
h.	**For those who <u>USED</u> PMTCT:** Do you remember when you received the (negative) baby's results? How did you feel? Why do you think your baby was negative? *Probe: was it PMTCT or something else?*
i.	For those who **<u>MISSED</u>** PMTCT: Did you know about PMTCT when you were pregnant? When did you find out you were HIV+? Why do you think you were not able to access PMTCT services when you were pregnant? *Probe: lack of knowledge/education, poor clinic access, personal choice, poor/absent clinic PMTCT services/commodities, did not believe it worked*
j.	For those who **<u>MISSED</u>** PMTCT: How do you think we could have educated you/convinced you to access PMTCT back then, to prevent your child from getting HIV?
k.	For those who **<u>MISSED</u>** PMTCT: From what you now know about PMTCT, do you think it works? Would you advise anyone who is HIV+ and pregnant to use PMTCT now?
l.	**All:** What do think these PMTCT services (drugs and testing) are supposed to do for the mother and baby? *Probe: how will we know when PMTCT is working very well?*
**III. Self-Perception of Mentor Mothers**<br>Section Introduction: *The purpose of the next section is to gather information about your views on the Mentor Mother in PMTCT*	
	a. **For those who are not MM:** Have you ever heard of mentor mothers (MM)? Can you describe who they are? Have you worked with them before, or have they helped you before?<br>b. **For M2M only:** have you ever wanted to be a mentor mother? Why or why not?
	c. **All:** Do you think MM can make a difference for the mother-infant pair in PMTCT? In other words, do you think using a MM can make things even better for the mother and baby with HIV? Why or why not? Please describe.
IV. **Users'/patients' opinions regarding quality of care and providing feedback.** *The next section asks questions about how patients are involved in the delivery of PMTCT services.*	
	a. What do you think about ANC clinic opening and closing time for ANC, PMTCT and delivery services? In other words, do you think the clinics are open when needed most? Probe: Please explain why you think so.
	b. Are you able to complain or praise the clinic when something is wrong, or if they do well? How are you able to complain or praise? Do you think the clinic listens to you? How do you think we can let patients provide information to the clinics on how they are doing?

**VI. General Perspective Questions**<br>Section Introduction: *The purpose of the last section is think about the PMTCT program in your LGA and state, and to tell us how you think we can improve HIV prevention services to women their babies.*
a. How do you think your local government and state are performing as far as PMTCT programs are concerned? Do you think they are reducing HIV transmission from mothers to their babies in this state/LGA? What are they doing well? What are they not doing well?
b. What changes would you suggest for your state/LGA/community's PMTCT program?
c. Do you think women with HIV get treated differently from HIV negative women at ANC/delivery? How so, and to what extent is the treatment different? Do you think it is different at your community/state compared to other communities/states?
d. What do you think the district or state government should do to prevent HIV/AIDS from spreading in your community? Please describe.
e. How well do you think we are working in HIV compared to other health issues like malaria, nutrition, immunization etc? Probe: do you think we are working harder on other health problems? Or do you think we are working much harder in HIV than other health problems?

Thank you for participating in this interview. We greatly appreciate your time and effort.
