# Supplementary material for: Acceptability of mentor mother peer support for women living with HIV in North-Central Nigeria: a qualitative study
Source: BMC Pregnancy Childbirth. 2021 Aug 7;21:545. doi: 10.1186/s12884-021-04002-1 (PMC8349095; doi:10.1186/s12884-021-04002-1)
Supplement: Supplementary file 5 — Additional file 5. IDI guide for PMTCT providers (formal health workers, traditional birth attendants, government officials/policy-makers, program implementers). [file 12884_2021_4002_MOESM5_ESM.pdf]

IHVN INSPIRE MoMent PMTCT Study

IHVN: Provider Group: Key Informant Interview

Interviewer's Name:

Date: Day/Month/Year

Participant Institution Group (eg State or Federal Ministry of Health, health facility, NGO), and Occupation:

Introduction:

The purpose of this key informant interview is to gather data from the informant about:

- *Challenges in PMTCT implementation, access, service delivery and retention*
- *Suggestions for how these challenges may be solved*
- *Acceptability of a Mentor Mother program in community*
- *Suggestions for how a Mentor Mother program may best be implemented*

The information we collect will only be reported together with information collected from other interviews. The names of informants will not be connected to the information collected.

I. Background

Section Introduction: *The purpose of the first section of the interview is to understand your background and role in PMTCT.*

Are you a doctor, nurse, community health extension worker, public health official, traditional birth attendant, program implementer, etc?

Do you work for a state, clinic, or NGO?

How long have you worked in your current position?

Please describe your role and responsibilities within PMTCT.

II. Assessment of PMTCT Implementation

Section Introduction: *The purpose of the next section of the interview is to understand activities conducted during the implementation phase of the PMTCT.*

a. Please describe, in your own understanding, how PMTCT programs are supposed to be implemented, as far as your institution or community is concerned.
b. At your institution, do PMTCT staff meet regularly to discuss program implementation challenges and successes? If yes, please describe how? If no, please comment on why these meetings are not held, or are not held regularly.

c. From your perspective, what factors contribute to HIV appointment and drug treatment adherence? *Probe: personal, provider, community factors*

d.	What happens if a patient is found to be non-adherent in terms of clinic visits or compliance with their medication regimen? How is this addressed (within your specific program)?
e.	Please describe the process of developing a case management plan under PMTCT? Probe- is this done as part of a case review with other staff? For example, are the cases discussed by a team of staff weekly? Who is usually present during these meetings?
f.	Please describe the process of follow up care for an HIV-positive woman. Who is clinically responsible for follow up case reviews? Probe: at your supported sites? At your particular facility? Is it a team? Individual doctor or nurse?
g.	How is the community educated/informed about the PMTCT services you provide?
h.	Please describe how the staff responds to the religious and cultural needs/concerns of the patients? Probe: for example, patient wanting female caregiver, preferring to deliver at home because it is first child, etc.
i.	What incentives or counseling are offered through the PMTCT services to help the patients follow up with recommended medical services and adhere to HIV medications?
j.	In your opinion, what would you say are the expected outcomes for PMTCT patients (mother and infant pairs)? Probe: How would you define PMTCT program success?

III. Perception of Mentor Mothers

Section Introduction: *The purpose of the next section is to gather information about your views on the Mentor Mother roles in PMTCT*

a.	Have you ever heard of mentor mothers (MM)? Can you describe who they are? Have you worked with them before?
b.	Do you think MM can make a difference in outcomes for the mother-infant pair in PMTCT? Why or why not? Please describe.

IV. **Self-perception of patients' opinions regarding quality of care and providing feedback.**

The next section asks questions about how patients are involved in the delivery of PMTCT services.

a.	What do you think are the opinions of patients regarding clinic hours for ANC, PMTCT and delivery services? In other words, how do you think patients feel about the availability of services when they need them? Probe: Please explain why you think so.
b.	(For staff working at clinic site only) What do you feel patients think about the services at your particular site? Have any of them told you personally, what they think about the services here?
c.	Do patients have opportunities to provide feedback about their experience (positive or negative) in the program? What exactly are these opportunities? If yes, how is this information used? If no, please comment on your opinion about the feedback process in general.

V. Your perceived role in providing PMTCT services

a. Do you feel that your work/program has been effective in implementing PMTCT programs (in terms of coverage/access, quality of services, retention)? Do you feel other providers, states/NGOs/sites are effective in their roles in providing PMTCT services? *Probe: What factors contribute to effectiveness? What factors decrease effectiveness?*

b. How important would you say your role is to the success of the program?

VI. General Perspective Questions

Section Introduction: *The purpose of the last section is to provide you with an opportunity to reflect on the PMTCT program and to provide your opinion about the successes and challenges you have experienced working to improve outcomes for patients.*

a. In your opinion, what are the strengths of your (state, NGO, facility) PMTCT program in reducing HIV transmission from the mother to her infant?

b. What changes would you suggest for your PMTCT program (national, state, NGO , or facility)?

c. Do you think women with HIV get treated differently from HIV negative women at ANC/delivery ? How so, and to what extent is the treatment different? Do you think it is different at your site/state compared to other sites/states?

d. In your view, what are the risk/behavior prevention activities needed to address HIV/AIDS in your community/the communities in which you work? Please describe.

On a scale of 0 -5, how would you rate your specific PMTCT program compared to other health programs in your state/site/NGO? eg malaria, nutrition, immunization ***0 is poor and 5 is excellent.___________***

Thank you for participating in this interview. We greatly appreciate your time and effort.
