# Supplementary material for: Acceptability of mentor mother peer support for women living with HIV in North-Central Nigeria: a qualitative study
Source: BMC Pregnancy Childbirth. 2021 Aug 7;21:545. doi: 10.1186/s12884-021-04002-1 (PMC8349095; doi:10.1186/s12884-021-04002-1)
Supplement: Supplementary file 6 — Additional file 6. IDI questionnaire for PMTCT facilitators: community leaders. [file 12884_2021_4002_MOESM6_ESM.pdf]

INSPIRE MoMent PMTCT Study

IHVN: Facilitators Group: In-Depth Interview

Interviewer's Name:

Date: Day/Month/Year

Participant specific group: ☐ Community Leaders

Introduction: *The purpose of this interview is to gather data from the informant about:*

- *Challenges in PMTCT implementation, access, service delivery and retention*
- *Suggestions for how these challenges may be solved*
- *Acceptability of a Mentor Mother program in community*
- *Suggestions for how a Mentor Mother program may best be implemented*

The information we collect will only be reported together with information collected from other interviews. The names of informants will not be connected to the information collected.

I. Background

Section Introduction: *The purpose of the first section of the interview is to understand your knowledge and experience with PMTCT.*

What is your background as a community leader? In what aspects do you provide leadership in your community?

When did you first hear about PMTCT? (Month and year) How did you hear about it?

What was your reaction/feeling when you first heard about PMTCT? Do you feel the same way now?

Where do you, your family, community go to access PMTCT services? (eg primary healthcare facility, General Hospital, private?)

II. Assessment of PMTCT Implementation

Section Introduction: *The purpose of the next section of the interview is to understand how you feel about the way we are providing PMTCT to the community.*

a. Please describe your best experience with PMTCT (or the best information you heard about PMTCT-if you have never used it).
b. Now please describe your worst experience with PMTCT services (or worst you have heard of).
c. Please describe, in your own understanding, how you think PMTCT services should be provided to the community. *Probe: what do you think we are doing well? What do you think we are not doing well? How can we improve?*

d. Why do you think that some people with HIV miss their appointments and do not take their drugs properly? *Probe: personal, provider, site, community factors*

e. What do you think the clinics should do if someone is not taking their drugs or misses their appointments? *Probe: do you think we should call them or send someone to their home?*

f. What do think these PMTCT services (eg drugs and testing) are supposed to do for the mother

and baby? *Probe: how will we know when PMTCT is working very well?*
**III. Perception of Mentor Mothers**<br>Section Introduction: *The purpose of the next section is to gather information about your views on the Mentor Mother in PMTCT*
Have you ever heard of mentor mothers (MM)? Can you describe who they are? Have you worked with them before, or have they helped you before?
Do you think MM can make a difference for the mother-infant pair in PMTCT? In other words, do you think using a MM can make things even better for the mother and baby with HIV? Why or why not? Please describe.
IV. O**pinions regarding quality of care and providing feedback.** *The next section asks questions about how you/patients are involved in the delivery of PMTCT services.*
a. What do you think about ANC clinic opening and closing time for ANC, PMTCT and delivery services? In other words, do you think the clinics are open when needed most? Probe: Please explain why you think so.
b. Are you able to complain or praise the clinic when something is wrong, or if they do well? How are you able to complain or praise? Do you think the clinic listens to you? How do you think we can let patients provide information to the clinics on how they are doing?
**VI. General Perspective Questions**<br>Section Introduction: *The purpose of the last section is think about the PMTCT program in your district and state, and to tell us how you think we can improve HIV prevention services to women their babies.*
a. How do you think your local government and state are performing as far as PMTCT programs are concerned? Do you think they are reducing HIV transmission from mothers to their babies in this state/district? What are they doing well? What are they not doing well?
b. What changes would you suggest for your state/district/community's PMTCT program?
c. Do you think women with HIV get treated differently from HIV negative women at ANC/delivery? How so, and to what extent is the treatment different? Do you think it is different at your community/state compared to other communities/states?
d. What do you think the district or state government should do to prevent HIV from spreading in your community? Please describe.
e. Now, let's focus on what you think <u>you</u> can do. As a community leader yourself, what do you think you can do to improve HIV prevention services to women and their babies? Probe: What do you think would help you do this better? What would limit you from making a difference?
f. Do you think we are working hard on HIV compared to other health issues like malaria, nutrition, immunization etc? Probe: do you think we are working harder on other health problems? Or do you think we are working much harder in HIV than other health problems?

Thank you for participating in this interview. We greatly appreciate your time and effort.
