# Supplementary material for: Acceptability of mentor mother peer support for women living with HIV in North-Central Nigeria: a qualitative study
Source: BMC Pregnancy Childbirth. 2021 Aug 7;21:545. doi: 10.1186/s12884-021-04002-1 (PMC8349095; doi:10.1186/s12884-021-04002-1)
Supplement: Supplementary file 7 — Additional file 7. COREQ checklist. [file 12884_2021_4002_MOESM7_ESM.docx]

**Consolidated criteria for reporting qualitative studies (COREQ) checklist for manuscript titled:**

***Acceptability of mentor mother peer support for women living with HIV in North-Central Nigeria:***

***A qualitative study***

| **No.** | **Item** | **Guide Questions/Description** | **Response (and Location of Relevant Narrative in Manuscript where applicable)** |
| --- | --- | --- | --- |
| **Domain 1: Research Team and Reflexivity** | | | |
| Personal Characteristics | | | |
| 1 | Interviewer/facilitator | Which author/s conducted the interview or focus group? | MAM, SE, NASA (female), LJC and AJA (male), two other female and three other male facilitators conducted the focus groups and In-depth interviews. |
| 2 | Credentials | What were the researcher’s credentials? E.g. PhD, MD | MAM (RN-MPH), SE (MPH), AJA (MPH), LJC (PhD), NASA (MD) |
| 3 | Occupation | What was their occupation at the time of the study? | NASA, Senior Technical Advisor Pediatric HIV, and Principal Investigator  MAM and SE, Research Associates  LJC, Social Science Professor  AJA, Program Officer, PMTCT |
| 4 | Gender | Was the researcher male or female? | Female authors: NASA, SE, MAM. Male authors: LJC and AJA. Overall, data analysis team comprised five females and five males including MAM, SE, NASA, AJA and LJC. (Materials and Methods- Data Transcription and Analysis) |
| 5 | Experience and training | What experience or training did the researcher have? | NASA (Trained; plus 4 years of conducting and analyzing qualitative studies in study setting and over 10 years of research experience)  MAM (Trained; plus 3 years of conducting and analyzing qualitative studies and surveys in study setting)  SE (Trained; plus 3 years of conducting and analyzing qualitative studies and surveys in study setting)  LJC (over 25 years’ experience in teaching, conducting, analyzing and publishing qualitative studies)  AJA (Trained; plus 6 months of conducting and analyzing qualitative studies and surveys in study setting) |
| Relationship with participants | | | |
| 6 | Relationship established | Was a relationship established prior to study commencement? | No, there was no established relationship with participants prior to study commencement beyond recruitment activities. |
| 7 | Participant knowledge of the interviewer | What did the participants know about the researcher? e.g. personal goals, reasons for doing the research | During orientation to study and consent process, researchers introduced themselves, stating where they worked (with an NGO/university and not for the health facility or government), and reasons for doing the research, namely, to improve the quality of health services to women and children living with, or affected by HIV. |
| 8 | Interviewer characteristics | What characteristics were reported about the interviewer/facilitator? Eg Bias, assumptions, reasons and interests in the research topic | Characteristics of interviewers and objectives are presented above and/or in the manuscript.  LJC is a male Social Scientist interested in the psychology and social determinants of health and disease among populations of African descent.  The Principal Investigator, NASA is a female pediatric infectious disease specialist interested in the prevention and treatment of HIV among African children.  MAM is a female nurse, Nigerian, multilingual, familiar with the religious and cultural context of study setting and interested in health and socio-economic inequalities disproportionately affecting women and children.  SE is a female public health professional, Nigerian, multilingual, and interested in maternal and child health and health promotion and prevention.  AJA is a male doctor, Nigerian, multilingual, familiar with the religious and cultural context of the study setting and interested in prevention and control of HIV, reproductive health and public health programming.  The three other male and three other female facilitators of the FGDs and IDIs were all Nigerian and multilingual, and each had between 2 and 20 years of experience conducting qualitative research in Nigeria and internationally. |
| **Domain 2: Study Design** | | | |
| Theoretical Framework | | | |
| 9 | Methodological orientation and Theory | What methodological orientation was stated to underpin the study? e.g. grounded theory, discourse analysis, ethnography, phenomenology, content analysis | Grounded theory with thematic analysis. (Materials and methods- Data transcription and analysis) |
| Participant selection | | | |
| 10 | Sampling | How were participants selected? e.g. purposive, convenience, consecutive, snowball | Purposive sampling for all participants. (Materials and methods- Study population and recruitment procedures) |
| 11 | Method of approach | How were participants approached? e.g. face-to-face, telephone, mail, email | First by healthcare workers and community gatekeepers (through phone calls and in person) to ascertain interest, then those interested were approached by research team in person. (Materials and methods-Study population and recruitment procedures) |
| 12 | Sample size | How many participants were in the study? | A total of 118 participants in 9 FGDs and 31 IDIs (Results and Table 1. FGD and IDI participant characteristics) |
| 13 | Non-participation | How many people refused to participate or dropped out? Reasons? | Three participants did not show up for focus group discussions (one in m2m group; two in MM group). One MM did not show due to a scheduling conflict. Non-participation information on the other two no-shows is not available. |
| Setting | | | |
| 14 | Setting of data collection | Where was the data collected? e.g. home, clinic, workplace | The FGDs and IDIs were conducted in private spaces at health facilities or in the study community. (Materials and methods-Study population and recruitment procedures) |
| 15 | Presence of non-participants | Was anyone else present besides the participants and researchers? | No one else was present besides participants, facilitators, and observers. (Materials and methods-Study population and recruitment procedures) |
| 16 | Description of sample | What are the important characteristics of the sample? e.g. demographic data, date | Most study participants were female and married. (Results). Rest of study characteristics are in Table 1. FGD and IDI participant characteristics. |
| Data collection | | | |
| 17 | Interview guide | Were questions, prompts, guides provided by the authors? Was it pilot tested? | The interviewers used FGD and IDI guides to collect data. These are attached in the Appendix. (Materials and methods- Data collection). The guides were not pilot-tested. |
| 18 | Repeat interviews | Were repeat interviews carried out? If yes, how many? | No, repeat interviews of participants were not conducted. |
| 19 | Audio/visual recording | Did the research use audio or visual recording to collect the data? | FGDs and IDIs were audio-recorded, and later transcribed. There were no visual recordings. (Materials and methods- Data collection). |
| 20 | Field notes | Were field notes made during and/or after the interview or focus group? | Yes, an observer made field notes during the FGDs to augment data analysis and interpretation. (Materials and methods-Data collection) |
| 21 | Duration | What was the duration of the interviews or focus group? | Each FGD lasted 1 ½ to 2 hours, and each IDI took approximately 45 minutes. (Materials and Methods- Data Collection) |
| 22 | Data saturation | Was data saturation discussed? | Thematic saturation was discussed and reached in the course of the FGDs and IDIs. (Materials and methods- Data transcription and analysis). |
| 23 | Transcripts returned | Were transcripts returned to participants for comment and/or correction? | No. Many of the participants could either not read or write or were not literate enough to be able to read and understand the transcripts regardless of whether they were written in English or local language. A verbal member check was not performed either. However the diverse and context-experienced facilitators and analyst team and our iterative analysis approach provided robust interpretation. |
| **Domain 3: Analysis and Findings** | | | |
| Data analysis | | | |
| 24 | Number of data coders | How many data coders coded the data? | Overall analysis was manually performed by a panel of ten paired researchers, (including MAM, SE, NASA and LJC and the rest of the facilitator team. AO (DrPH, MPhil Sociology & Development studies) additionally performed independent analysis  (Materials and methods-Data transcription and analysis) |
| 25 | Description of the coding tree | Did authors provide a description of the coding tree? | In initial coding, identified code words/phrases were categorized in hierarchical fashion into a coding tree, with parent (main) codes under which related subcategory codes were arranged. Ultimately, categories were combined or expanded to represent emergent themes. (Materials and Methods- Data Transcription and Analysis) |
| 26 | Derivation of themes | Were themes identified in advance or derived from the data? | We used the constant comparison method of Grounded Theory in a thematic analysis approach to derive themes from the data.  (Materials and Methods- Data Transcription and Analysis) |
| 27 | Software | What software, if applicable, was used to manage the data? | All transcripts were manually analyzed. No specialized qualitative software was used to manage the data.  (Materials and methods- Data transcription and analysis) |
| 28 | Participant checking | Did participants provide feedback on the findings? | No. Member checks were not performed. |
| Reporting | | | |
| 29 | Quotations presented | Were participant quotations presented to illustrate the themes / findings? Was each quotation identified? e.g. participant number | Participant quotations are presented and identified by participant group and number (Throughout Results section) |
| 30 | Data and findings consistent | Was there consistency between the data presented and the findings? | Yes, discussion on findings was written to align with results/data presented |
| 31 | Clarity of major themes | Were major themes clearly presented in the findings? | Major themes, such as Acceptability of MMs, role of MMs, MM service delivery approach, and stigma and discrimination, are clearly presented in the Results section and in Figure 1. |
| 32 | Clarity of minor themes | Is there a description of diverse cases or discussion of minor themes? | Yes, minor themes and/or divergent cases were discussed under each of the major themes. The needs of MMs (training, remuneration and validation as lay workers) and non-disclosure of HIV-status to clients by a few MMs was a minor theme identified. Some respondents had divergent views on the acceptability of work props, especially HIV identifying attire and ID cards. (Results) |
